# Association between parent attitudes and receipt of human papillomavirus vaccine in adolescents

**DOI:** 10.1186/s12889-017-4787-5

**Published:** 2017-10-02

**Authors:** Jeffrey J. VanWormer, Casper G. Bendixsen, Elizabeth R. Vickers, Shannon Stokley, Michael M. McNeil, Julianne Gee, Edward A. Belongia, Huong Q. McLean

**Affiliations:** 1 Center for Clinical Epidemiology and Population Health, Marshfield Clinic Research Institute, 1000 North Oak Ave, Marshfield, WI 54449 USA; 2 Centers for Disease Control and Prevention, Atlanta, USA

**Keywords:** Human, Papillomavirus vaccines, Parents

## Abstract

**Background:**

Human papillomavirus (HPV) vaccine coverage rates remain low. This is believed to reflect parental hesitancy, but few studies have examined how changes in parents’ attitudes impact HPV vaccine uptake. This study examined the association between changes in parents’ vaccine attitudes and HPV vaccine receipt in their adolescent children.

**Methods:**

A baseline and 1-year follow-up survey of HPV vaccine attitudes was administered to parents of 11–17 year olds who had not completed the HPV vaccine series. Changes in attitudinal scores (barriers, harms, ineffectiveness, and uncertainties) from the Carolina HPV Immunization Attitudes and Beliefs Scale were assessed. Two outcomes were measured (in parents’ adolescent children) over an 18-month period and analyzed using multivariable regression; receipt of next scheduled HPV vaccine dose and 3-dose series completion.

**Results:**

There were 221 parents who completed the baseline survey (11% response rate) and 164 with available follow-up data; 60% of their adolescent children received a next HPV vaccine dose and 38% completed the vaccine series at follow-up. Decrease in parents’ uncertainties was a significant predictor of vaccine receipt, with each 1-point reduction in uncertainties score associated with 4.9 higher odds of receipt of the next vaccine dose. Higher baseline harms score was the only significant predictor of lower series completion.

**Conclusions:**

Reductions in parents’ uncertainties appeared to result in greater likelihood of their children receiving the HPV vaccine. Only baseline concerns about vaccine harms were associated with lower series completion rate. Education for parents should emphasize the HPV vaccine’s safety profile.

## Background

The human papillomavirus (HPV) vaccine was introduced in 2006, but coverage has remained lower than for other recommended adolescent vaccines for tetanus, diphtheria, pertussis (Tdap), and meningococcal disease [1]. HPV vaccine series completion, which included three doses in 2015, was recently estimated at 35% in the U.S. Factors contributing to low population-level HPV vaccine coverage are not well understood. Parents are generally aware of the HPV vaccine [2], but specific knowledge about HPV vaccination (e.g., schedule, benefits) is weakly correlated with actual coverage [3]. While multiple causes likely exist, low coverage is hypothesized to be a function of hesitancies, ambivalence, or resistance by many parents who are deciding about their child’s healthcare. In considering their adolescent’s young age and (presumed) timeline until sexual exposure, parents typically underestimate their child’s susceptibility to acquiring sexually transmitted infections or cervical cancer [4, 5]. Parents also tend to overestimate the risks of HPV vaccine adverse events [4, 6, 7], some of which are real (e.g., syncope) [8], while causal links to others have been repudiated (e.g., thromboembolism [9], risky sexual behaviors [10]) despite widely available false claims.

The degree to which parents’ HPV vaccine attitudes influence their decisions to have their adolescent children get the HPV vaccine series has been examined in prior studies [11–13]. Most prior investigations, however, were cross-sectional and assessed self-reported vaccination or vaccine intent. There are few longitudinal studies that have examined how HPV vaccine attitudes influence HPV vaccine series completion, particularly those that take advantage of more objective vaccination data sources such as medical records. The purpose of this study was to examine the association between changes in parents’ HPV vaccine attitudes and HPV vaccine uptake in their adolescents, using electronic health records (EHR) data from a north-central Wisconsin integrated healthcare system. The hypothesis was that positive changes in parental attitudes would be associated with greater adolescent HPV vaccine uptake.

## Methods

### Design and participants

This was a cohort analysis of survey data, conducted in parallel with a medical care quality improvement project designed to increase HPV vaccine coverage in adolescent patients from seven Wisconsin communities who receive care from Marshfield Clinic Health System (MCHS; headquarters Marshfield, WI) [14]. Briefly, intervention activities included medical department-level education and support, individualized provider feedback on adolescent vaccination coverage in their patient panel, and initiation of HPV vaccine reminder/recall notices mailed to parents of 12 year old adolescent children. Interventions were focused on medical providers in the outpatient primary care environment, and did not target parents or the general public directly. Parental HPV attitudes were surveyed at baseline and one year later in the intervention communities. Study eligible individuals completed both the baseline and follow-up attitudinal survey and were parents of adolescents who, at baseline: were medically homed to one of the nine MCHS regional centers (in the seven target communities) selected for the intervention, were aged 11–16 years, had failed to start or complete the HPV vaccine series (i.e., <3 vaccine doses), and were able to respond to the English language survey. Criteria used to assign each adolescent’s medical home included their named primary care provider at a given medical center; ≥1 preventive care visit there in the past year, or ≥2 qualifying visits (Evaluation and Management visit types) for diagnosis and treatment within the past three years.

### Survey procedures

Survey-eligible parents were selected using stratified random sampling by community. Baseline surveys were distributed over three months in Spring 2015, with the follow-up survey administered one year later. Contact information for eligible adolescents was extracted from MCHS administrative records and an invitation was mailed to the “Parents of” each enumerated adolescent. The mailing contained a cover letter describing the study and an invitation to participate, as well as the survey instrument and a postage-paid return mailer. A reminder letter was sent to non-respondents about one month after the initial mailing, and a phone outreach was also made to remaining non-respondents. A passive consent procedure was utilized whereby participants were informed that completed surveys would be linked to their EHR data. A $2 cash incentive was enclosed in all mailed follow-up survey invitations. Study procedures were approved by the Marshfield Clinic Institutional Review Board.

### Measures

The primary predictor was change between baseline and 1-year follow-up in the sub-factor scores from the Carolina HPV Immunization Attitudes and Beliefs Scale (CHIAS) [15]. Attitudinal changes were accounted for by subtracting each participant’s follow-up CHIAS (sub-factor) score from their baseline CHIAS score. The CHIAS is a 16-item instrument that includes four sub-factor scores for perceived barriers, harms, effectiveness, and uncertainties related to the HPV vaccine. Each item has Likert-scale response options scored from 1 to 4 points, with a mean score generated for each of the four sub-factors. To improve interpretation, a slight modification was made to the effectiveness sub-factor in that responses were reverse ordered and it was renamed ‘ineffectiveness’ so that, for all sub-factor scores, higher values corresponded to less favorable attitudes/endorsement of the HPV vaccine. As done previously [15], CHIAS items with missing responses were imputed with sample mean values. Also, for those with multiple HPV vaccine eligible adolescent children, parents were asked to only consider their youngest adolescent child when formulating their baseline survey responses, and to again consider that same adolescent child for their follow-up survey responses.

Two HPV vaccine outcomes were examined in separate analyses, and these variables again pertained to each parent’s youngest HPV vaccine eligible adolescent child. These HPV vaccine outcomes were assessed over 18 months of follow-up after each participant completed their baseline survey. The first outcome was receipt of the next (scheduled) HPV vaccine dose, which captured those who received their first (i.e. initiation), second, or third (i.e., series completion) dose (depending on how many HPV vaccine doses were received before baseline). The second outcome was receipt of all three doses of HPV vaccine (i.e., 3-dose series completion, the standard of care during this study timeframe). Data on vaccinations were collected from a regional population-based immunization registry [16], which is sourced by EHR and other data (e.g., public health agencies, schools).

Several covariates were also analyzed that were suspected of possibly modifying or confounding the association between changes in parents’ HPV attitudes and adolescent HPV vaccine receipt. From the baseline survey, this included each parent’s age, gender, education level (high school or less, some college, Associate degree, Bachelor’s degree, Graduate degree), health insurance coverage (private, public-assisted, none), and baseline CHIAS scores. From the follow-up survey, this included report of a physician’s recommendation to get the HPV vaccine in the prior year. In addition, EHR data on each parent’s youngest adolescent child’s prior HPV, Tdap, and meningococcal vaccination history, number of ambulatory care visits over three years (prior to baseline), gender, and age were also examined as covariates.

### Analysis

Sociodemographic characteristics were reported descriptively. Logistic regression was used to examine the association between changes in parents’ HPV vaccine attitudes and receipt of the HPV vaccine in their adolescent child. Basic models were first created to examine the crude associations between each CHIAS sub-factor change score (follow-up score minus baseline score) and HPV vaccine receipt. Next, given the well documented influence of physician advice on HPV vaccination [17–19], a test for effect modification was entered into the basic model by creating two-way interaction terms between each CHIAS sub-factor change score and physician’s recommendation to get the HPV vaccine in the prior year. Any interaction terms with a significant (*p* < 0.05) association with HPV vaccine receipt were retained in subsequent models. Finally, a reduced multivariable model was fit by adding each covariate separately and applying forward selection to exclude any covariates that were not significant independent predictors of HPV vaccine receipt. The two HPV vaccine receipt outcomes were tested separately using this same analytical approach. All analytical procedures were conducted using SAS Version 9.4 (Cary, NC).

## Results

At baseline, 1998 surveys were mailed to eligible parents, with 221 (11%) respondents. Among those, 187 returned responses at the 1-year follow-up, with 175 confirmed as the same parent who responded at baseline (cohort retention 79%). Eleven respondents were excluded from analyses due to missing covariate data, yielding a final analytical sample of 164 parents. Parent respondents were primarily Non-Hispanic White females (Table 1). Because surveys were mailed to the parents of study-eligible adolescent patients (and any parent/guardian could respond), the enumerated sample was not identifiable. Thus parent information (e.g., age, sex) only became available upon survey completion, which precluded comparisons between invited parents who did vs. did not respond to the survey. Comparisons were possible on some adolescent characteristics though. Relative to non-responders, study-eligible adolescents of parents who responded to the survey were significantly younger and more likely to have received Tdap and meningococcal vaccines. There was no difference in the proportion of adolescents who received a prior HPV vaccine.

**Table 1:** Baseline characteristics of parents of North-Central Wisconsin adolescents without a completed human papillomavirus vaccine series, separated by characteristics of parents and their adolescent child

	Survey respondents^a^ *n* = 164	Survey non-respondents *n* = 1777	*p*
Parent Characteristics			
Mean age (years)	43.7 ± 6.1	NA	
Gender		NA	
Female	136 (83%)		
Male	28 (17%)		
Race/ethnicity		NA	
White, Non-Hispanic	157 (96%)		
Not White or Hispanic	5 (3%)		
Unknown	2 (1%)		
Education		NA	
High school or less	13 (8%)		
Some college	23 (14%)		
Associates degree	40 (24%)		
Bachelors degree	48 (29%)		
Graduate degree	40 (24%)		
Health insurance		NA	
Private	137 (83%)		
Public-assisted	26 (16%)		
Unknown	1 (1%)		
Adolescent Characteristics			
Age			0.041
11–13 years	118 (72%)	1137 (64%)	
≥ 14 years	46 (28%)	640 (36%)	
Gender			0.083
Female	79 (48%)	732 (41%)	
Male	85 (52%)	1045 (59%)	
Mean number of ambulatory care visits over past 3 years	4.2 ± 2.6	3.9 ± 2.9	0.182
Received 1–2 prior HPV vaccine doses			0.921
Yes	53 (32%)	581 (33%)	
No	111 (68%)	1196 (67%)	
Received Tdap and meningococcal vaccines			0.001
Yes	142 (87%)	1385 (78%)	
No	22 (13%)	392 (22%)	

^a^Values are reported as frequency (% of column total) or mean ± SD

At baseline, 32% of parents’ adolescent children had initiated the HPV vaccine, having received 1–2 prior HPV vaccine doses (none had completed the 3-dose series, per study eligibility criteria). Sixty percent of parents’ adolescent children (58% of males, 62% of females) had received at least one HPV vaccine dose and 38% (44% of males, 33% of females) had completed the 3-dose series by the end of the 18-month follow-up period. As outlined in Table 2, the highest baseline CHIAS sub-factor scores were for harms (mean ± SD = 2.2 ± 0.6 points) and uncertainties (2.2 ± 0.7 points), followed by ineffectiveness (2.1 ± 0.9 points), and barriers (1.4 ± 0.4 points). Barriers and harms scores remained stable over time. Scores for uncertainties and ineffectiveness improved, both decreasing by 0.2 points (~10% improvement; *p*’s < 0.001) over one year. At the 1-year follow-up survey, 77% of parents reported having received a physician’s recommendation for their adolescent child to get the HPV vaccine in the prior year.

**Table 2:** Baseline, 1-year follow-up, and change in Carolina HPV immunization attitudes and beliefs scale (CHIAS) sub-factor scores among parents of North-Central Wisconsin adolescents without a completed human papillomavirus vaccine series

	Baseline^a^	Follow-up	Change (follow-up minus baseline points)
CHIAS scores (1–4 points)			
Barriers	1.4 ± 0.4	1.4 ± 0.4	0.0 ± 0.4
Harms	2.2 ± 0.6	2.1 ± 0.6	0.0 ± 0.5
Uncertainties	2.2 ± 0.7	2.0 ± 0.7	−0.2 ± 0.6
Ineffectiveness	2.1 ± 0.9	1.8 ± 0.9	−0.2 ± 0.7

^a^Values are reported as mean ± SD

### Next HPV vaccine dose

The basic models revealed that changes between baseline and follow-up in CHIAS sub-factor scores for harms, uncertainties, and ineffectiveness were significantly associated with receipt of next HPV vaccine dose. Parents whose attitudes became more favorable over time had a higher likelihood of vaccine receipt. Specifically, each 1-point decrease in the harms, uncertainties, or ineffectiveness score was associated with 5.4 ([95%] CI: 2.0, 14.1), 3.8 (CI: 1.9, 7.5), and 2.3 (CI: 1.3, 4.2) higher odds of having received the next HPV vaccine dose, respectively. After covariate adjustment, most associations with attitudinal change scores were attenuated, leaving only the CHIAS uncertainties change score, baseline CHIAS uncertainties score, and baseline CHIAS harms score as significant predictors of receipt of next HPV vaccine dose (Table 3). Prior physician’s recommendation to get the HPV vaccine was not found to be an effect modifier, but was retained in the final multivariable model as a significant independent predictor, along with health insurance coverage and receipt of a prior HPV vaccine dose at baseline.

**Table 3:** Basic and multivariable logistic regression models of the association between human papillomavirus (HPV) vaccine receipt and Carolina HPV immunization attitudes and beliefs scale (CHIAS) sub-factor scores, along with covariates, among North-Central Wisconsin parents (N = 164)

	Next HPV vaccine dose receipt (Yes vs. No)		HPV vaccine series completion (Yes vs. No)	
	Basic ^a^ OR (95% CI)	Multivariable OR (95% CI)	Basic OR (95% CI)	Multivariable OR (95% CI)
CHIAS change scores (follow-up minus baseline points)				
Barriers	1.6 (0.6, 4.1) *p* = 0.320	--- ^b^	1.9 (0.7, 5.1) *p* = 0.215	---
Harms	5.4 (2.0, 14.1) *p* < 0.001	---	2.8 (1.1, 6.8) *p* = 0.025	---
Uncertainties	3.8 (1.9, 7.5) *p* < 0.001	4.9 (2.0, 12.2) *p* < 0.001	2.3 (1.1, 4.5) *p* = 0.022	---
Ineffectiveness	2.3 (1.3, 4.2) *p* = 0.004	---	1.3 (0.8, 2.1) *p* = 0.334	---
CHIAS baseline scores (points at baseline)				
Harms	---	11.7 (4.0, 34.2) *p* < 0.001	---	5.2 (2.2, 12.1) *p* = 0.001
Uncertainties	---	2.5 (1.0, 6.2) *p* = 0.044	---	---
Health insurance^c^				
Public-assisted vs. private	---	6.0 (1.5, 23.6) *p* = 0.011	---	---
Provider recommended HPV vaccine				
Yes vs. no	---	4.1 (1.3, 12.9) *p* = 0.015	---	---
1–2 prior HPV vaccine doses				
Yes vs. no	---	7.1 (2.0, 25.6) *p* = 0.003	---	14.0 (5.8, 34.1) *p* < 0.001

^a^Values are reported as odds ratio (95% confidence interval, *p*-value), relative to the reference category for categorical variables or a 1-unit decrease for CHIAS scores, for the HPV vaccine outcomes

^b^--- Variable not considered in basic models or excluded from multivariable model

^c^Comparison between none vs. private health insurance was not estimable due to so few participants without health insurance coverage

### HPV vaccine series completion

The basic models for this analysis found changes between baseline and follow-up in CHIAS sub-factor scores for harms and uncertainties were significant predictors of HPV vaccine series completion. Similar to the previous analysis, parents whose attitudes improved had a greater likelihood of completing the HPV vaccine series. Each 1-point decrease in the harms or uncertainties score was associated with 2.8 (CI: 1.1, 6.8) and 2.3 (CI: 1.1, 4.5) higher odds of their adolescent’s series completion, respectively. And again, associations with attitudinal change scores were attenuated after covariate adjustment, with only baseline CHIAS harms score and receipt of prior HPV vaccine being significantly associated with HPV vaccine series completion during the follow-up period (Table 3).

## Discussion

Prior research has shown that parents’ CHIAS scores are significant predictors of their intent to get their child vaccinated against HPV, as well as their self-reported history of HPV vaccine receipt [15, 20, 21]. To our knowledge, this was the first study to examine how changes in parents’ attitudes were associated with improvements in objectively-measured HPV vaccine receipt. Despite relatively good uptake in HPV vaccine during the 18-month study timeframe, vaccine attitudes appeared to be less flexible in parents, as concerns about effectiveness and uncertainties waned modestly, while beliefs about barriers and harms did not change. In the multivariable model, only change in the CHIAS uncertainties score was significantly associated with receipt of the next dose of HPV vaccine. Parents who experienced a favorable shift in uncertainties had a greater likelihood of their adolescent child receiving at least one HPV vaccine dose during the follow-up period. No changes in CHIAS scores were associated with completion of the HPV vaccine series.

Though the baseline survey response rate was low and somewhat skewed toward parents of younger adolescents who were more apt to have received Tdap/meningococcal vaccines, parental concerns about the safety of the HPV vaccine seemed to be the most influential attitudinal barrier. On average, the CHIAS harms score remained stable between baseline and follow-up. But even among parents whose CHIAS harms score shifted favorably, their adolescents were not more likely to get the HPV vaccine after covariate adjustment. In contrast, the baseline CHIAS harms score was strongly associated with receipt of the next HPV vaccine dose and was the only significant attitudinal predictor of HPV vaccine series completion. Thus it seemed parents with high harms scores were not apt to change their minds, and even if they did, it had no impact on HPV vaccine receipt. This finding was consistent with some prior studies. Notably, the CHIAS harms score was also among the strongest predictors of HPV vaccine initiation after one year among parents of adolescent girls in North Carolina [21]. Also, fear of side effects was among the main reasons cited for avoiding HPV vaccination (or intent to vaccinate) in cross-sectional studies with Dutch [22] and Romanian parents [23]. This suggests that public awareness and patient education interventions should emphasize the HPV vaccine’s post-licensure safety profile [9]. How to best translate such messaging to a clinical environment, however, is yet unclear. Compared to brief/straightforward recommendations, more in-depth conversational approaches by medical providers may yield limited influence on parents’ decision-making regarding HPV vaccination [24, 25].

Several covariates were also significantly associated with HPV vaccine receipt. As expected, a prior HPV vaccine dose was the strongest predictor of both receipt of next dose and series completion. A reported physician’s recommendation to get the HPV vaccine in the prior year was also associated with receiving the next HPV vaccine dose, but adolescents’ age and gender were not associated with receipt of next dose or series completion. Interestingly, parents with publicly-assisted health insurance had six times greater odds of their adolescent child initiating or getting their next HPV vaccine dose during the follow-up period relative to parents with private health insurance. Reasons for this are obviously speculative, but it may be related to some Medicaid pay-for-performance initiatives targeting childhood immunizations [26], recognizing that such incentives have had limited impact in other settings [27, 28]. Why public-assisted health insurance did not translate into greater likelihood of HPV vaccine series completion though was unclear, but could be related to fewer total preventive care visits in adolescents from lower income households [29].

This study was strengthened by the systematic sampling of parents of adolescents who receive primary care within an integrated regional healthcare system, a level at which clinical care quality improvement initiatives are apt to occur. Also, adolescents’ clinical data on HPV vaccine coverage was linked to their parents’ CHIAS scores. In terms of limitations, the baseline survey response rate was of particular concern because it was very low, which might reflect sensitivities about the general topic of HPV or the lack of a baseline response incentive. Low baseline response does not negate the associations observed within the cohort and is not by itself indicative of nonresponse bias [30, 31], but it can increase the potential for selection biases if respondents were very different from non-respondents. Unfortunately, a comparison between parents who did vs. did not respond to the survey was impossible since only those who responded could be identified from the enumerated sample. The adolescent children of parent respondents vs. non-respondents, however, were significantly younger and more likely to have received Tdap/meningococcal vaccine, but not HPV vaccine. The assumption in this analysis was that changes in parents’ HPV vaccine attitudes affected their decision to have their adolescent child get the HPV vaccine. But the temporality of the attitudinal exposures (or time when measured) and HPV vaccination period overlapped some, thus reverse causation could be influential to a degree in that some parents’ HPV attitudes measured at the 1-year follow-up may have only shifted after (and as a function of) their adolescent child received the HPV vaccine. Other study limitations included the limited generalizability of our sample, which was relatively small and selected from a racially homogenous source population.

## Conclusion

HPV vaccine coverage remains low in the U.S. [1], but improving parents’ HPV vaccine attitudes is considered a key factor in increasing these low rates [32]. How flexible some parents’ HPV vaccine attitudes are, or how responsive to targeted intervention they may be, remains uncertain. Though the baseline response rate was low in this sample of Wisconsin parents, attitudes about the HPV vaccine’s effectiveness and certainty in having their adolescent child get it improved modestly. Reduced uncertainties scores over one year was a significant predictor of adolescent children getting their next scheduled HPV vaccine dose, while baseline harms score (which, on average, did not improve over time) was the main attitudinal factor associated with HPV vaccine series completion. Given the positive influence of medical providers’ advice on parents’ HPV vaccine decisions in prior studies [17–19], as well as our current study, the healthcare system appears to be an optimal setting to test future methods to improve HPV vaccine attitudes and coverage.

## Abbreviations

CHIAS: Carolina HPV Immunization Attitudes and Beliefs Scale
CI: Confidence Interval
EHR: Electronic Health Records
HPV: Human Papillomavirus
MCHS: Marshfield Clinic Health System
OR: Odds ratio
Tdap: tetanus, diphtheria, and pertussis
